# Analysis of MiR-20b, MIR-197 markers for differentiation between forensic body fluids encountered in sexual assault cases

**DOI:** 10.1007/s12024-024-00831-6

**Published:** 2024-06-10

**Authors:** Shimaa Ahmed Alsaeed, Noha Maher Elrewieny, Rabab Abdulmoez Amin Eltokhy, Mohamed Shokr Mohamed, Wagdy K. B. Khalil, Aziza B. Shalby, Hoda F. Booles, Heba Mohamed Aboubakr

**Affiliations:** 1 Department of Forensic Medicine and Clinical Toxicology, Faculty of Medicine, Cairo University, Kasr Alainy Street, Cairo, 11562 Egypt; 2 Department of Andrology, Faculty of Medicine, Cairo University, Kasr Alainy Street, Cairo, 11562 Egypt; 3 Hormones Department, National Research Center, P.O. Box 12622, Dokki, Cairo, Egypt; 4 Department of Cell Biology, Biotechnology Research Institute, National Research Centre, P.O. Box 12622, Dokki, Cairo Egypt

**Keywords:** MiR-20b & MiR-197, Forensic body fluid identification, Vaginal fluid, Fertile & infertile semen

## Abstract

Identifying body fluids can be a critical clue that aids in reconstructing the crime scene. Semen and vaginal fluid identification is crucial, especially in cases of sexual assault. The majority of forensic studies focused on identifying normal body fluids and neglected the expression variation of semen in pathology. To differentiate between vaginal fluids, fertile and infertile semen samples (oligospermia and azoospermia) using miR 20b and miR197. A total of 48 body fluid samples, divided as 16 vaginal fluids, 16 fertile semen, and 16 infertile semen samples (8 with oligospermia and 8 with azoospermia), were collected, and the expression levels of miR-20b and miR-197 were detected by the SYBR Green real-time quantitative PCR technique. Our results showed significant different expression of these miRNAs in normal semen compared to vaginal and infertile semen. Moreover, we designed a model based on Fisher’s discriminant function to forecast the group affiliations of unidentified samples. With three novel equations, we were able to accurately distinguish between semen and vaginal fluid, fertile and infertile semen, and oligospermia and azoospermia semen samples with validation accuracy of 81.3%, 100%, and 100%, respectively. MiR-20b and miR-197 expression levels are efficient and appropriate markers to distinguish semen from vaginal fluid and to differentiate between fertile and infertile semen samples. However, the present study is a preliminary study based on clinical samples, and the potential role of these markers in differentiating real crime scene samples is still unknown, so we recommend further research to investigate these markers expression while using forensic samples.

**Supplementary Information:**

The online version contains supplementary material available at 10.1007/s12024-024-00831-6.

## Background

Identifying body fluids can provide critical clues that aid in reconstructing the crime scene and providing compelling evidence for the courts. Semen, vaginal fluids, menstrual and peripheral blood are among the body fluids that are commonly discovered at criminal scenes [1]. Semen and vaginal fluid identification is crucial in cases of sexual assault [2].

The approaches for identifying body fluids in criminal cases were broadened to include miRNA, DNA methylation, and mRNA [3, 4]. The non-coding single-stranded RNA class known as microRNAs has a standard length equal to 22 bases” with “of about 21–23 nucleotides” and is responsible for regulating the expression of genes in these organisms [5].

MicroRNAs are thought to have the potential to be effective tools for body secretion differentiation due to their great tissue sensitivity, outstanding stability, and remarkable preservation [6]. MiRNAs are currently being used in numerous studies to determine the origins of body fluids [3, 7, 8].

The specificity of such miRNAs has already been demonstrated in documented empirical research, which also looked at the possibility of their existence in deteriorated and aging tissues. But the majority of forensic studies were focused on identifying normal body fluids and neglected to take into account the expression variation of seminal disorders. Based on statistics, infertility due to male factors represents about one-half of the infertility issues that impact 6% of men worldwide. However, the molecular mechanisms underlying such disorders are still unexplained [9].

Forensic professionals continue to face the following questions: First, is the expression of fertile and infertile semen the same? If not, do these variations impair the ability to distinguish different body secretions from semen? Additionally, could infertile semen be identified using the same body fluid identification criteria and range? Could the semen be effectively distinguished by those miRNAs? [10].

Two miRNAs (miR-20p and miR-197) were chosen, in the present work, to construct an identification model attempting to differentiate between semen and vaginal secretions. Moreover, the efficacy of miR-20b and miR-197 expression differences will be assessed to distinguish between fertile and infertile semen samples with oligospermia (OS) and azoospermia (AZ).

## Materials and methods

The current work is a cross-sectional analytical study in which two miRNA markers (miR-20b and miR-197) have been selected to differentiate between semen and vaginal fluid and to evaluate their accuracy in distinguishing between fertile and infertile semen samples. It was conducted in the Forensic Medicine, Clinical Toxicology, and Andrology Departments, Faculty of Medicine, Cairo University, in cooperation with the Departments of Cell Biology and Biotechnology, National Research Centre. Written informed consent has been gathered from all volunteers before the study.

### Ethical approval

The scientific ethical committee of the Kasr Al-Ainy Faculty of Medicine at Cairo University gave its approval to the study with ethical approval number **N- 301**.

### Sampling

48 body fluid samples, divided as follows: 16 vaginal fluid, 16 fertile semen, and 16 infertile semen samples (8 with oligospermia and 8 with azoospermia), were collected from Egyptian adult (above the age of eighteen) healthy volunteers from both sexes, while single, pregnant women, individuals complaining of vaginal infection, abnormal vaginal discharge, history of chronic medical disorders, and the presence of infectious diseases such as HIV, HCV, and HBV, were excluded from our study. Semen samples were collected in a sterile plastic cup and liquefied at 7 °C for 30 min. Semen-free vaginal fluid samples were gathered by sterile cotton swaps, left to dry at ambient temperature for 24 h, and stored at -80 °C.

### RNA extraction

The standard TRIzol® Reagent Extraction Method (Invitrogen, Germany) was used to isolate total RNA from semen and vaginal smear samples.

### Reverse transcription (RT) reaction

The whole poly(A) + RNA extracted from semen cells and vaginal smear samples was reverse transcribed into cDNA in a total amount of 20 L using MystiCq® microRNA cDNA Synthesis Mix (Sigma-Aldrich; Merck KGaA).

### Real-time polymerase chain reaction (RT-PCR)

The StepOneTM Real-Time PCR System from Applied Biosystems (Thermo Fisher Scientific, Waltham, MA, USA) was used to estimate the semen cells and vaginal smear sample copy numbers. PCR reactions were set up in 25-L reaction mixtures containing 12.5 L of SYBR®-Green I GoTaq® qPCR Master Mix (Promega Corporation), 0.5 L of 0.2 M sense primer, 0.5 L of 0.2 M antisense primer, 6.5 L of distilled water, and 5 L of cDNA template. The sequences of specific primers for miR-20b and miR-197 with U6 as the internal control are listed in Table 1 [11, 12]. By using the 2CT method, the relative quantification of the target to the reference was established.

**Table 1:** Primers sequence used for microRNAs expression

Gene	Gene sequence
miR-20b	F: GCG CAA AGT GCT CAT AGT GC R: AGT GCA GGG TCC GAG GTA TT
miR-197	F: ATT ACT TTG CCC ATA TTC ATT TTG A R: ATT CTA GAG GCC GAG GCG GCC GAC ATG T
U6	F: CTC GCT TCG GCA GCA CA R: AAC GCT TCA CGA ATT TGC GT

F, forward; R, reverse

### Data analysis

Statistical software for the social sciences, SPSS version 28 (IBM Corp., Armonk, NY, USA), was used to code and enter the data. The mean and standard deviation were employed to summarize the data. Multiple comparisons in an analysis of variance (ANOVA) Quantitative factors were compared using the post-hoc Tukey test [13]. Starting with a test of the equality of means between semen and other fluids as well as between infertile and fertile semen, discriminant analysis was conducted. The discriminate function was determined using stepwise statistics, which identified the significant predictors. After that, group centroids (group means) were established; these serve as the key points for differentiating across groups. According to the discriminate function, the percentage of correctly categorized cases was categorized [14]. A P-value of 0.05 or lower represented significant statistical difference.

## Results

### The expression level of miR-20b and miR-197 in the studied samples

The relative expression alterations of miR-20b and miR-197 in the studied body fluids are demonstrated in Table 2. The mean values of expression levels of miR-20b showed statistically significant expression differences between all body fluid samples with a P-value < 0.001, where the mean expression level was the highest in fertile semen, followed by oligospermic semen, then azoospermic semen, and finally was the vaginal fluid with the lowest mean expression level.

**Table 2:** Relative expression alterations of miR-20b and miR-197 in the studied body fluids

	Fertile semen		Oligospermia		Azoospermia		Vaginal fluid		
	Mean	SD	Mean	SD	Mean	SD	Mean	SD	*P*-value
Relative expression of miR-20b	1^a^	0.11	0.47^b^	0.06	0.18^c^	0.05	0.07^d^	0.04	< 0.001
Relative expression of miR-197	1^a^	0.06	0.31^b^	0.04	0.09^c^	0.05	0.05^c^	0.02	< 0.001

Different superscript denotes significant differences

*P*-value < 0.05 is statistically significant

In the same trend, the expression levels of miR-197 showed the highest mean value in fertile semen, followed by oligospermic semen, azoospermic semen, and finally vaginal fluid. The decrease in the expression levels of miR-197 was statistically significant (P-value < 0.001) between the studied body fluids except between azoospermic semen and vaginal fluid, which showed a non-significant decrease.

**Fig. 1:**
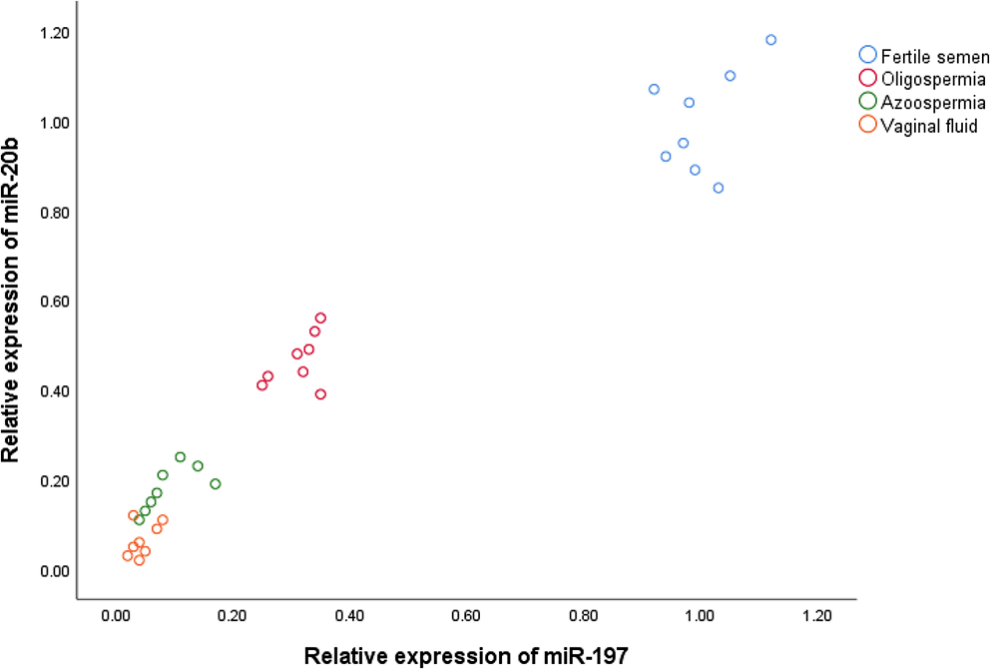
Two-dimensional (2D) scatter plot represented the expression range of miR-20b, miR-197 in fertile, oligospermic, azoospermic semen and vaginal fluid samples

The relative expression levels of miR-20b and miR-197 were employed to create a double-dimensional scatter plot, which displayed differential expression distribution in body fluid samples as illustrated in Fig. 1. Blue, red, green, and yellow circles represented the expression range of the miRNAs in fertile, oligospermic, azoospermic, and vaginal fluid samples, respectively.

### The fisher discriminant function between body fluid samples

In the present study, the data subsets were subjected to Fisher discriminant analysis to forecast the group identity of unknown body fluids. Three Fisher discriminant functions were created, after extensive calculations and analysis, using the relative expression levels of miR-20b and miR-197 as independent variables and fertile semen, infertile semen “azoospermia and oligospermia” and vaginal fluid samples as dependent variables.

#### Discriminant function analysis to identify semen from vaginal fluid

Y1 = 11.648* Relative expression of miR-20b-8.455* Relative expression of miR-197-1.921.

If Y1 > 0, a sample was identified as semen, and if Y1 < 0, it was classified as vaginal fluid. Self-validation and cross-validation were used to confirm the discriminant function’s accuracy, as illustrated in Table 3, where the accuracy rate of the function Y1 for the relevant data subset was 81.3%.

**Table 3:** Discriminant function’s classification accuracy between fertile semen and vaginal fluid samples

Classification Results^a, c^					
		Fluid	Predicted Group Membership		Total
			Fertile semen	Vaginal fluid	
Original	Count	Fertile semen	18	6	24
		Vaginal fluid	0	8	8
	%	Fertile semen	75.0	25.0	100.0
		Vaginal fluid	0.0	100.0	100.0
Cross-validated^b^	**Count**	Fertile semen	18	6	24
		Vaginal fluid	0	8	8
	%	Fertile semen	75.0	25.0	100.0
		Vaginal fluid	0.0	100.0	100.0

a. 81.3% of original grouped cases correctly classified

b. Cross validation is done only for those cases in the analysis. In cross validation, each case is classified by the functions derived from all cases other than that case

c. 81.3% of cross-validated grouped cases correctly classified

#### Discriminant function analysis to differentiate between fertile sand infertile semen

Y2=-6.113* Relative expression of miR-20b + 15.513* Relative expression of miR-197-3.904.

If Y2 > 0, a sample was considered to include fertile semen, and if Y2 < 0, it contained infertile semen. 100.0% of the cross-validated grouped instances were correctly classified, as demonstrated in Table 4.

**Table 4:** Discriminant function’s classification accuracy between fertile and infertile semen samples

Classification results^a, c^										
		semen fertility			Predicted Group Membership					Total
					Fertile semen		Infertile semen			
Original	Count		Fertile semen		8		0			8
			Infertile semen		0		16			16
			Ungrouped cases		0		8			8
	%		Fertile semen		100.0		0.0			100.0
			Infertile semen		0.0		100.0			100.0
			Ungrouped cases		0.0		100.0			100.0
Cross-validated^b^	Count		Fertile semen		8		0			8
			Infertile semen		0		16			16
	%		Fertile semen		100.0		0.0			100.0
			Infertile semen		0.0		100.0			100.0

a. 100.0% of original grouped cases correctly classified

b. Cross validation is done only for those cases in the analysis. In cross validation, each case is classified by the functions derived from all cases other than that case

c. 100.0% of cross-validated grouped cases correctly classified

#### Discriminant function analysis to identify infertile oligospermia from azoospermia samples

Y3 = 10.364 * Relative expression of miR-20b + 13.133* Relative expression of miR-197-6.

A sample was labeled as a semen sample with oligospermia if Y3 > 0 and as an azoospermia sample if Y3 < 0. Self-validation and cross-validation were shown in Table 5. The accuracy rate of the function Y3 was 100%.

**Table 5:** Discriminant function’s classification accuracy between oligospermia and azoospermia semen samples

Classification Results^a, c^										
				Oligospermia versus azoospermia		Predicted Group Membership			Total	
						Oligospermia		Azoospermia		
Original		Count		Oligospermia		8		0	8	
				Azoospermia		0		8	8	
				Ungrouped cases		8		8	16	
		%		Oligospermia		100.0		0.0	100.0	
				Azoospermia		0.0		100.0	100.0	
				Ungrouped cases		50.0		50.0	100.0	
Cross-validated^b^		Count		Oligospermia		8		0	8	
				Azoospermia		0		8	8	
		%		Oligospermia		100.0		0.0	100.0	
				Azoospermia		0.0		100.0	100.0	

a. 100.0% of original grouped cases correctly classified

b. Cross validation is done only for those cases in the analysis. In cross validation, each case is classified by the functions derived from all cases other than that case

c. 100.0% of cross-validated grouped cases correctly classified

## Discussion

Numerous biological processes, such as cell division and differentiation, depend significantly on miRNAs. They are highly conserved across species, extremely stable, and prevalent in seminal plasma and various body secretions [3, 15]. miRNAs were found to be highly abundant in semen; however, it is important to note that different methods of investigations may produce different results [16–19].

In the current study, two miRNA markers (miR-20b and miR-197) were selected, and their relative expression levels were applied to distinguish between semen and vaginal secretion as well as to study their accuracy to differentiate between fertile and infertile semen (oligospermia and azoospermia) clinical samples. The expression levels of miR-20b and miR-197 showed significant expression differences between all body fluid samples with a P-value < 0.001, but the expression levels of miR-197 in azoospermia samples were relatively similar to those in vaginal fluid without significant differences.

We designed a model based on Fisher’s discriminant function to differentiate various bodily fluid samples using miR-197 and miR-20b markers. The findings can be directly derived using expression data and a mathematical formula, avoiding some subjectivity and producing reliable and accurate results. With three novel equations, we were able to accurately distinguish between fertile semen & vaginal fluid, fertile & infertile semen and between oligospermia & azoospermia samples with validation accuracy of 81.3%, 100% and 100%, respectively.

Weber et al [20] studied the expression level of miRNAs in 12 human body fluids and stated that miR-197 and miR-20b are among the top 20 most prevalent miRNAs in semen samples, while the miRNA species that are uniquely detected in semen fluids are miR-197, miR-20b, miR-20b miR-380, miR-29b-2, miR-508-5p, miR-340, miR- 644, miR-17, miR-588, miR-617, and miR-1. They also demonstrated that miR-10a, miR-135b, miR-135a, and miR-10b have co-expression with semen in different body fluids.

MiR-891a and miR-888 were discovered by other researchers to have a uniform and focused distribution. These markers are able to successfully distinguish semen from a variety of forensically relevant body fluids with great semen specificity [16–18, 21]. However, according to the findings of Tong et al. [19] and Hanson et al. [22], there was overlapping in the expression of miR-891a regarding normal semen and various bodily fluids.

He et al. [6] developed a statistical model for classifying five human body fluids on the basis of the different expression levels of 10 miRNAs (miR-451a, miR-214-3p, miR-144-3p, miR-205-5p, miR-144-5p, miR-654-5p, miR-888-5p, miR-891a-5p, miR-203-3p, and miR-124a-3p) in semen, saliva, vaginal secretions, saliva, peripheral blood, and menstrual blood. A discriminant function has been created using stepwise discriminant analysis, and the model’s accuracy in self-validation, cross-validation, identification validation set accuracy, and blind test result accuracy were all 100%.

As regards identification of infertile semen samples, in the research done by Tian et al. [10], the level of expression of different sets of miRNA markers (miR-10a, miR-10b, miR-135b, miR-888, miR-135a and miR-891a) has been assessed in normal semen as well as four different types of infertile semen samples: (asthenospermia, oligospermia, azoospermia, and asthenospermia) using real-time quantitative PCR. They stated that these markers have significant high expression in normal semen and the model’s self-validation accuracy was 100%.

Tian et al [23] investigated the expression levels of two miRNAs (10b and 135b) in various types of semen from infertile males, including asthenospermia and azoospermia and the results revealed that fertile semen expressed substantially more of the two miRNAs than did the asthenospermia and azoospermia samples. Additionally, Haas et al. [24] studied the expression of the semen miRNA markers (PRM2 and PRM1) in fertile and azoospermic semen and they stated that it was negative in azoospermic samples.

Our results were sufficient to demonstrate the existence of a difference in the expression of the studied markers between semen and vaginal fluid, also between fertile and infertile semen and between oligospermia and azospermia semen clinical samples. Moreover, this study is the first to examine the efficacy of miR-197 and miR-20b to distinguish between fertile & infertile semen and between azoospermia & oligospermia semen samples and few studies, to our knowledge, have examined the significant effect of semen fertility problems on other miRNAs markers. We could therefore introduce the use of these miRNAs markers in the field of body fluid differentiation. However, this study is based on clinical samples, thus, further studies are needed using real forensic samples from crime scenes to investigate if these markers expression can provide significant forensic body fluid identification when such samples are found in different crime scenes as in sexual assault cases.

## Conclusion

MiR-20b and miR-197 expression levels are efficient and appropriate markers to distinguish semen from vaginal fluid and to differentiate between fertile and infertile semen samples. However, the present study is a preliminary study based on clinical samples and the potential role of these markers in differentiation of real crime scene samples is still unknown, so we recommend further research to investigate these markers expression while using forensic samples.

**Key points**

1. Semen and vaginal fluid identification is crucial in criminal investigations.

2. MiR-20b and miR-197 expression levels can differentiate semen from vaginal fluid.

3. These markers? expression levels distinguish between fertile & infertile semen.

4. These markers? expression levels distinguish between azoospermia & oligospermia.

## Electronic supplementary material

Below is the link to the electronic supplementary material.

Supplementary Material 1
