# Supplementary material for: Analysis of MiR-20b, MIR-197 markers for differentiation between forensic body fluids encountered in sexual assault cases
Source: Forensic Sci Med Pathol. 2024 Jun 10;21(1):56–62. doi: 10.1007/s12024-024-00831-6 (PMC11953183; doi:10.1007/s12024-024-00831-6)
Supplement: Supplementary file 1 — Supplementary Material 1 [file 12024_2024_831_MOESM1_ESM.docx]

**Highlights**

1. Semen and vaginal fluid identification is crucial in criminal investigations.
2. MiR-20b and miR-197 expression levels are effective to identify semen from vaginal fluid and to identify infertile semen.
3. Three novel equations based on Fisher's discriminant analysis were obtained to distinguish between semen & vaginal fluid, fertile & infertile semen, and oligospermia & azoospermia semen samples with validation accuracy of 81.3%, 100%, and 100%, respectively.
4. We were the first to examine the efficacy of miR-197 and miR-20b to distinguish between fertile & infertile semen and between azoospermia & oligospermia semen samples.
